# Perineural Invasion Should Be Regarded as an Intermediate-Risk Factor for Recurrence in Surgically Treated Cervical Cancer: A Propensity Score Matching Study

**DOI:** 10.1155/2021/1375123

**Published:** 2021-08-03

**Authors:** Ting Wan, Hua Tu, Lili Liu, He Huang, Yanling Feng, Jihong Liu

**Affiliations:** ^1^Department of Gynecologic Oncology, Sun Yat-sen University Cancer Center, State Key Laboratory of Oncology in South China, Collaborative Innovation, Center for Cancer Medicine, Guangzhou, China; ^2^Department of Pathology, Sun Yat-sen University Cancer Center, State Key Laboratory of Oncology in South China, Collaborative Innovation, Center for Cancer Medicine, Guangzhou, China

## Abstract

**Background:**

Perineural invasion (PNI) is considered as a poor prognostic factor in cervical cancer, but there has been no postoperative adjuvant therapy for it, because whether it belongs to high- or intermediate-risk factors has not been determined, this study intends to provide evidences to solve this problem.

**Methods:**

We conducted a retrospective analysis of cervical cancer patients who underwent radical surgery and be reported PNI from January 2012 to June 2017 at the Sun Yat-sen University Cancer Center. After 1 : 1 propensity score matching (PSM), a group of patients without PNI was matched according to the clinical pathological features. Postoperative pathological parameters and prognosis were evaluated between the PNI and the matched groups.

**Results:**

1836 patients were screened, of which 162 (8.8%) diagnosed as stages IB1 to IIB reported PNI. Comparing to the matched group, more PNI (+) patients had deep outer cervix stromal invasion, cervical tunica adventitia invasion, positive lymph nodes, and positive margins. Among patients without high-risk factors, PNI (+) patients had worse 3-year overall survival (90.8% vs. 98.1%, *P* = 0.02), PNI (+) patients with single intermediate-risk factor and PNI (-) patients who meet with SEDLIS criteria had similar progress free survival (*P* = 0.63) and overall survival (*P* = 0.63), even similar survival curves.

**Conclusion:**

PNI is related to a worse overall survival among cervical cancer patients without high-risk factors and play the role as an intermediate-risk factor.

## 1. Introduction

Perineural invasion (PNI) is the neoplastic invasion of nerves by cancer cells and is an important prognostic factor related to poor outcomes in many malignancies such as head and neck and digestive tract carcinomas [1–4]. Numerous studies have explored the mechanism of PNI, and suggested that it could be the fourth route of cancer spread [5–7]. Nevertheless, the roles of PNI in cervical cancer have not been paid enough attention by gynaecologists, although many pathology centers have made routine reports about PNI in postoperative pathological results. A few studies have discussed the prognostic value of PNI in cervical cancer, but came to contradictory conclusions [8–13]. In 2015, a systematic review and meta-analysis suggested that PNI is a poor prognostic factor for cervical cancer, but only three small sample retrospective cohorts were included in the study and failed to distinguish whether PNI is a high- or intermediate-risk factor.

According to the clinical practice guidelines of the National Comprehensive Cancer Network (NCCN), there are definite recommendations that patients should receive adjuvant radiation if they have any of the following high-risk factors after radical surgery: lymph node metastasis, parametrial invasion, or resection margin involvement. Intermediate-risk factors have also been defined as lymphovascular space invasion (LVSI), stromal invasion, and tumour size according to the Sedlis criteria [14]. However, PNI has not been recognized as an intermediate- or high-risk factor in cervical cancer. While postoperative pathology reports of many patients illustrate PNI conditions, there are currently no treatment recommendations for PNI.

Therefore, we conducted this retrospective and matched cohort study with the largest sample size in the literature and intent to demonstrate that PNI is a high-risk factor or intermediate-risk factor for cervical cancer.

## 2. Material and Methods

This study has been approved by the appropriate Ethics Committee and Institutional Review Board (IRB) of the Sun Yat-sun University Cancer Center (SYSUCC, Guangzhou, China) in 2020, and the requirement for written informed consent was waived by the IRB because no specific privacy of the patients was involved. Since 2012, PNI was gradually recognized and thus reported at SYSUCC as part of the patient's pathological results. Diagnosis of PNI was defined: surgically resected cervical cancer tissues were fixed in 10% neutral buffered formaldehyde fixative and embedded in paraffin, and the specimens were cut into 4 *μ*m thick sections and stained with hematoxylin and eosin. When microscopic examination revealed that cancer cells infiltrated any layer of nerve fibers (including epineurium, perineurium, and endoneurium) or surrounded nerve circumference ≥33%, it was judged as PNI positive. When the pathologist fails to confirm whether the tissue was an invaded nerve bundle, the immunohistochemical staining specific marker S100 was used to help determine [15]. Data from all patients who underwent radical hysterectomy and pelvic lymphadenectomy at SYSUCC between January 2012 and June 2017 were assessed. Patients were screened if PNI has been reported in the postoperative pathological results and were diagnosed according to the International Federation of Gynaecology and Obstetrics (FIGO, 2009) as stage IA2 to IIB squamous cell carcinoma, adenocarcinoma, or adenosquamous carcinoma of the cervix. Other subtypes such as neuroendocrine or clear cell carcinoma was excluded in this study. Patients who had a cone biopsy or definite radiation before radical surgery were also excluded because of the possibility of missed diagnosis of PNI. Neoadjuvant chemotherapy with 2-3 cycles of the paclitaxel and cispaltin regimen could apply in patients with stage IB2, IIA2, or IIB prior to radical surgery.

After surgery, patients with any of the high-risk factors such as lymph node metastasis, parametrial invasion, and resection margin involvement would undergo adjuvant concurrent chemoradiation with weekly cisplatin, whereas patients with any two of the following intermediate factors would receive adjuvant radiation (modified Sedlis criteria): pathological tumour size larger than 4 cm, deep stromal invasion, and lymphovascular space invasion (LVSI). The volume of adjuvant radiation included parametrium, vaginal margin, presacral nodes, the entirety of the common iliac, external iliac, internal iliac, and obturator nodes. In patients with common iliac and/or para-aortic nodal involvement, extended field pelvic and para-aortic radiotherapy up to the level of renal vessels was delivered. The dose was approximately 50 Greys (Gy) with conventional fractionation of 1.8 to 2.0 Gy daily; additional brachytherapy with a dose of 18 to 21 Gy was given to patients who had positive vaginal stump. Finally, patients with one intermediate-risk factor received 2-3 cycles adjuvant chemotherapy of paclitaxel and cisplatin or underwent observation, which is according to the will of patients.

The clinical and pathological characteristics of the patients were recorded, including age, FIGO stage, tumour size, histological type, tumour differentiation, preoperative treatment, surgical approach, postoperative adjuvant therapy, LVSI, ovarian invasion, lower uterine segment invasion, deep stromal invasion of the cervical canal, deep stromal invasion of the cervix, lymph node invasion, parametrium invasion, positive margins, and PNI.

The follow-up data were also recorded. Follow-up procedures included physical examination, abdominal and pelvic ultrasonic examination, serum squamous carcinoma cell antigen level (for squamous carcinoma and adenosquamous carcinoma), and CA125 (for adenocarcinoma and adenosquamous carcinoma). These were performed in SYSUCC every three months for two years and then every six months until the fifth year. Computed tomography (CT) or magnetic resonance imaging (MRI) scans were performed when doctors suspected recurrence.

**Figure 1:**
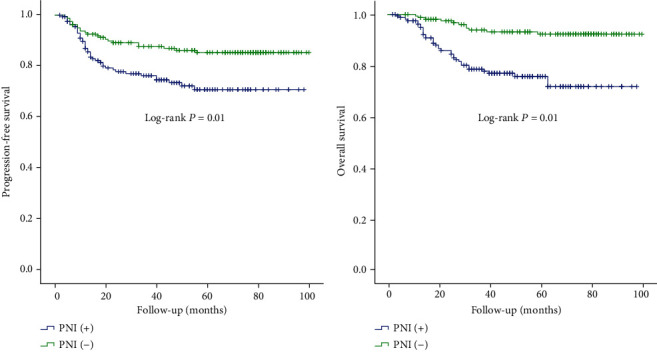
Progression-free survival and overall survival of patients in group PNI (+) and PNI (-).

According to the literature, if the pathological slides are not reread with special staining, the real incidence of PNI may be underestimated [11]. The PNI situation we collected from the pathological record may not represent the real PNI population, while the workload of rereading all the cervical cancer pathological slides from 2012 to 2017 is very huge. In order to avoid this bias, we matched a group of PNI (-) patients according to baseline clinical characteristics, including tumour size, pathological type, FIGO stage, tumour differentiation, and preoperative treatment in the same period by using 1 : 1 propensity score matching (PSM), and the pathological slides of matched group were reread by the pathologist to confirm there was no PNI. The match tolerance of PSM was set to 0.01. Propensity scores of individuals were calculated using logistic regression analysis (SPSS version 23.0, Chicago, IL, USA), baseline characteristics were analysed using Chi-square statistics or Fisher's exact test in the case of categorical variables, and the *t*-test or analysis of variance (ANOVA) for continuous variables. The flow diagram of recruitment and exclusion is shown in Figure 1S (Supplementary materials).

The comparisons of characteristics between two groups were performed using nonparametric statistics. Numerical parameters were expressed as the median and range, and the Mann-Whitney *U* test was used to examine the differences. Fisher's exact test and the Chi-square test were used to compare rates between two groups, as appropriate. Survival curves were constructed according to the Kaplan-Meier estimator, and differences were compared using the log-rank test. A two-tailed *P* value < 0.05 was considered statistically significant. All statistical analyses were performed using the SPSS software version 23.0 (SPSS Inc, Chicago, IL, USA).

## 3. Results

### 3.1. Patients Characteristics

Between January 2012 and June 2017, 1836 patients staged from IA2 to IIB received radical hysterectomy and pelvic lymphadenectomy with or without para-aortic lymph node dissection. Of them, 162 patients (8.8%) reported PNI after surgery, and the FIGO stage of these patients ranged from IB1 to IIB. No patients with stage IA were reported PNI.  Table 1 summarizes the patients' demographics and tumour features assessed before and after PSM. Most PNI were found in the cervix, 10 patients had PNI in the parametrium or surgical margin, and two patients had found PNI in the metastatic ovaries.

The mean age of patients with PNI was 51.5 ± 9.0 years old. The average tumour size was 3.9 ± 1.3 cm. The histological type was mostly squamous cell carcinoma, but adenocarcinoma or adenosquamous cell carcinoma accounted for 28.4%, which was larger than that in the normal cervical cancer population. 95.1% of patients had tumours with moderate or poor differentiation. Furthermore, 92.6% of patients underwent open radical hysterectomy, whereas only 7.4% received minimally invasive surgery.

After 1 : 1 PSM, there were no significant differences in pathological type, tumour differentiation, FIGO stage, primary therapy, NACT courses, and surgical approach between two groups. However, patients in the matched group were younger (50.8 vs. 51.5 years, *P* = 0.05), and more patients had pathological complete remission or complete remission after NACT (20.3% vs. 0%, *P* = 0.01).

### 3.2. Postoperative Characteristics and Adjuvant Therapy

Table 2 demonstrates the postoperative characteristics of patients with PNI and its matched group. No significant differences were observed between two groups in terms of LVSI, ovarian invasion, and invasion of the lower uterine segment. However, more patients with PNI had deep cervical canal stromal invasion (66% vs. 39.5%, *P* = 0.01), deep outer cervix stromal invasion (90.7% vs. 61.1%, *P* = 0.01), cervical tunica adventitia invasion (45.1% vs. 10.5%, *P* = 0.01), positive lymph nodes (35.2% vs. 18.3%, *P* = 0.01), and positive margins (37% vs. 14.8%, *P* = 0.01).

Since the patients with PNI had more risk factors than patients in the matched group, instances of postoperative adjuvant therapy were also higher: 80.2% of PNI patients underwent adjuvant radiation with or without chemotherapy, whereas the ratio in the matched group was 69.1%.

### 3.3. Survival Outcomes

#### 3.3.1. Progression-Free Survival and Overall Survival of All Patients

The last follow-up date was 1st October 2020, and the median follow-up period was 55 months (range, 2-100 months). Recurrence was observed in 40 patients with PNI, with a median time for recurrence at 12 months. For the matched group, there were 22 patients and 12 months, respectively. Moreover, 27 patients with PNI (27/40, 67.5%) and 10 patients in the matched group (45.5%) had distant recurrence with or without local recurrence, which means larger ratio of patients in PNI group had distant recurrence (*P* = 0.03). After treatment for recurrence, 36 patients (90%) with PNI and 11 patients (50%) in the matched group eventually died (*P* = 0.01).

The 3-year progression-free survival (PFS) rate for patients with PNI and those in the matched group was 76.4% (95% confidence interval (CI): 72.9%-79.9%) and 87.8% (95% CI: 72.2%-90.4%) (*P* = 0.01), whereas the 3-year overall survival (OS) rate was 78.6% (95% CI: 75.2%-82.0%) and 93.9% (95% CI: 91.9%-95.9%) (*P* = 0.01), respectively. The PFS and OS survival curves for these two groups are represented in Figure 1.

#### 3.3.2. PFS and OS of Patients Stratified by High-Risk Factors

**Figure 2:**
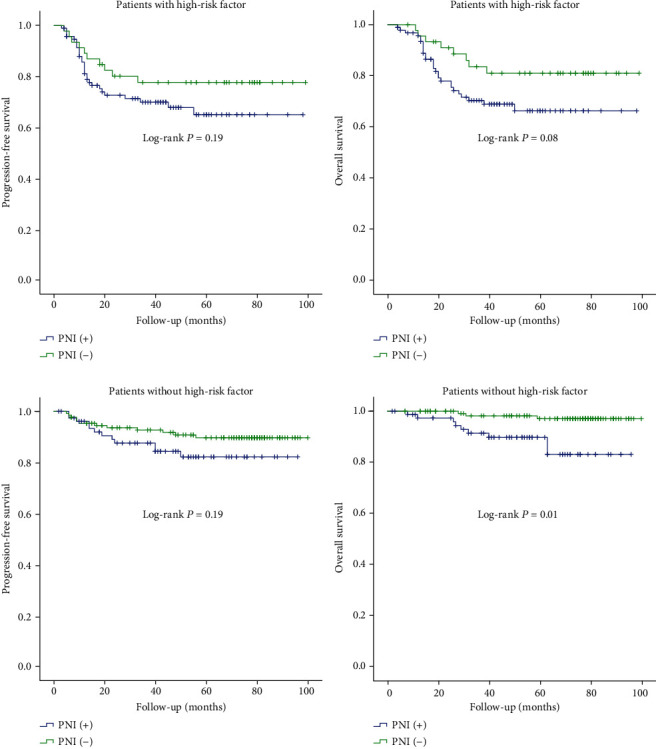
Progression-free survival and overall survival of patients in PNI (+) and PNI (-) patients stratified by high-risk factors (any of the following factors: positive lymph node, positive parametrium, or positive surgical margin).

In order to understand the exact impact of PNI in different risk subgroup, we stratified the patients by the high-risk factors (with any of these three factors: positive lymph nodes, parametrium, or margin) and observed their PFS and OS. In the PNI and matched groups, 89 (54.9%) and 43 (26.5%) patients had one or more high-risk factors, respectively. Among patients with high-risk factors, we could see a worse trend in patients with PNI, the 3-year PFS for patients with PNI and those in the matched group was 67.9% (95% CI: 62.7%-77.4%) and 76.2% (95% CI: 69.6%-82.8%) (*P* = 0.19), and the 3-year OS was 68.3% (95% CI: 63.0%-73.6%) and 82.4% (95% CI: 76.4%-88.4%) (*P* = 0.08), respectively. On the other hand, among patients without high-risk factors, the 3-year PFS for patients with PNI and those in the matched group was 86.6% (95% CI: 82.4%-90.8%) and 92.1% (95% CI: 89.6%-94.6%) (*P* = 0.14), and the 3-year OS was 90.8% (95% CI: 87.2%-94.4%) and 98.1% (95% CI: 96.7%-99.5%) (*P* = 0.02), respectively (Figure 2), indicating that PNI play an important role on poor prognosis in patients without high-risk factors.

#### 3.3.3. PNI (+) Patients with One Moderate-Risk Factor vs. PNI (-) Patients Who Met with Sedlis Criteria

**Figure 3:**
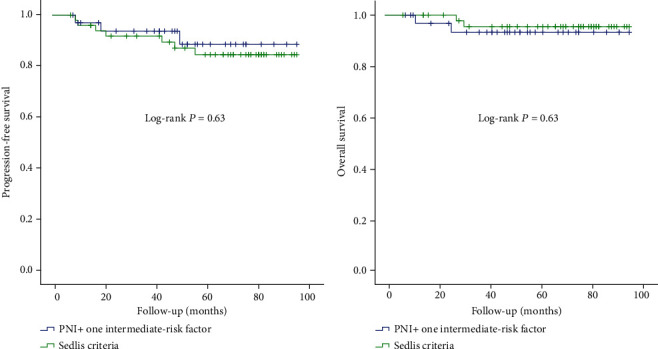
Progression-free survival and overall survival in patients with PNI + one intermediate-risk factor and patients who meet with Sedlis criteria but without PNI.

To further confirm whether PNI is an intermediate-risk factor, we picked out the PNI (+) patients who combined with single intermediate-risk factor (any of the following factors: tumor size large than 4 cm, deep stromal invasion, or LVSI) and those PNI (-) patients who met with Sedlis criteria (intermediate-risk factors from NCCN guideline). There were 34 PNI (+) patients combining with single intermediate-risk factor, three of them recurred and two died; 50 PNI (-) patients met with the Sedlis criteria, seven of them recurred, and two died. The survival curve of these patients is shown in Figure 3, indicating a similar PFS (*P* = 0.63) and OS (*P* = 0.63), even the similar survival curves (Figure 3). We proved that PNI might be a new intermediate-risk factor who play a similar role in cervical cancer like large tumor size, deep stromal invasion, or LVSI.

## 4. Discussion

PNI is an important prognostic factor in many malignancies, which is also the indication for adjuvant therapy. However, in cervical cancer, there is still controversy about whether PNI is an independent prognostic factor. In the studies reported by Elsahwi et al. [11] (192 patients included; 24 had PNI) and Cho et al. [12] (185 patients included; 13 had PNI), PNI was not associated with worse prognosis in early cervical cancer. By contrast, in the study reported by Tang et al. [16] (larger cohort, 406 patients included; 43 had PNI), PNI were identified as independent risk factors for OS and DFS. None of past studies has been able to state with certainty whether PNI is a high- or intermediate-risk factor. In our matched case study, we firstly proved that PNI was an intermediate-risk factor systematically. As the original finding of intermediate-risk factors for cervical cancer, any single intermediate-risk factor has not been definitely proved an independent poor prognostic factor, but pairwise combinations of the intermediate-risk factors can be shown to clearly affect prognosis.

The factors associated with PNI are clear enough in the literature, which were LVSI, deep stromal invasion, tumour size ≥ 4 cm, and parametrium invasion [8–13, 17]; we also found these common features in our cohort. What is interesting is that we specifically found a relatively higher proportion of patients who had adenocarcinoma or adenosquamous cell carcinoma with the moderate or poor differentiation, and these findings were seldom reported in the past studies. In 2019, Wang et al. conducted a multi-institutional Chinese cohort to explore the reproducibility and prognostic significance of Silva pattern system in adenocarcinoma of cervical cancer, which is still a new pattern for the pathology of adenocarcinoma. They found perineural invasion was significantly correlated with the Silva pattern system and appeared in most Silva C tumors (*P* = 0.001), and they suggested revising the Silva C criteria by adding perineural invasion as a factor [18]. These characteristics can help us better understand and predict PNI.

Tumour metastasis along nerve has been proven by clinical researches. Capek et al. reviewed 17 cases of bladder, rectal, and cervical cancers and concluded that as tumour spreads, with parts of the nerve invasion confirmed by biopsy, the L5-S1 spinal nerves and the sciatic nerve were most frequently involved, and tumour cells could use the splanchnic nerves as conduits and spread from the end organ to the lumbosacral plexus [19]. In our study, we found that PNI could present in the ovaries or the surgical margin, away from the local tumour of the cervix. According to the recommendations of NCCN guidelines, for patients with FIGO stage IB1-IIA2 cervical cancer, the recommended surgical procedure is type C radical hysterectomy (Querleu-Morrow classification system), involving two subtypes: type C1 and type C2, of which type C1 is also known as Nerve-Sparing Radical Hysterectomy (NSRH). However, the detail indication for NSRH has not been established, which may not be suitable for patients prone to PNI according to the existed evidences. Concurrent chemoradiotherapy might be the proper treatment for the patients prone to PNI rather than NSRH, and clinical trials should be carried out to verify these considerations.

Our study possibly has the largest sample size of cervical cancer patients with PNI (among English language published data); we provide meaningful information about the new clinical characteristics, exact prognostic value, and further treatment for patients with PNI which were all seldom mentioned in the past studies. Nevertheless, our study may have two possible weaknesses. First, we did not review all the pathological slides of the 1836 patients to identify the actual incidence rate of PNI, and this may leave out a small number of patients with insignificant PNI. The review of all the slides was an enormous workload that we could not realistically finish; nevertheless, the data we used were based on the reported pathological results and were in accordance with the actual clinical situation. Second, the matched cases may not entirely represent the population of PNI (-) cases. However, we evaluated the clinical characteristics and prognosis of the patients in matched group, and concluded that these patients were in line with our general clinical cognition of normal population in the same FIGO stages.

## 5. Conclusions

PNI is likely to occur in cervical cancer patients with risk factors. Our study firstly proved that PNI play the role as an intermediate-risk factor when without high-risk factors, further studies should explore the adjuvant therapy for PNI.

## Figures and Tables

**Table 1:** Patient characteristics.

Patient characteristics		Before PSM			After PSM		
		PNI (+), *n* = 162	PNI (-), *n* = 1674	*P*	PNI (+), *n* = 162	PNI (-), *n* = 162	*P*
Mean age (yr)		51.5 ± 9.0	50.3 ± 9.6	0.03	51.5 ± 9.0	50.8 ± 9.1	0.05

Average tumor size (cm)		3.9 ± 1.3	2.6 ± 1.6	0.01	3.9 ± 1.3	3.9 ± 1.2	0.93

Pathological type	Squamous carcinoma	116 (71.6%)	1462 (87.3%)	0.02	116 (71.6%)	118 (72.8%)	0.89
	Adenocarcinoma	31 (19.1%)	153 (9.1%)		31 (19.1%)	28 (17.3%)	
	Adenosquamous carcinoma	15 (9.3%)	59 (3.5%)		15 (9.3%)	16 (9.9%)	

Tumor differentiation	Well	8 (4.9%)	235 (14.0%)	0.01	8 (4.9%)	6 (3.7%)	0.93
	Moderate	63 (38.9%)	724 (43.3%)		63 (38.9%)	63 (38.9%)	
	Poor	91 (56.2%)	715 (42.7%)		91 (56.2%)	93 (57.4%)	

FIGO stage	IA2	0	63 (3.8%)	0.01	0	0	0.96
	IB1	41 (25.3%)	752 (44.9%)		41 (25.3%)	38 (23.5%)	
	IB2	13 (8.0%)	209 (12.5%)		13 (8.0%)	14 (8.6%)	
	IIA1	59 (36.4%)	204 (12.2%)		59 (36.4%)	60 (37.0%)	
	IIA2	26 (16.1%)	136 (8.1%)		26 (16.0%)	27 (16.7%)	
	IIB	23 (14.2%)	98 (5.9%)		23 (14.2%)	23 (14.2%)	

Primary therapy	Radical surgery	111 (68.5%)	1284 (76.7%)	0.01	111 (68.5%)	98 (60.5%)	0.12
	NACT	51 (31.5%)	390 (23.3%)		51 (31.5%)	64 (39.5%)	

NACT courses		2.3	2	0.36	2.3	2.2	0.78

Response to NACT	pCR	0	41 (10.5%)	0.01	0	6 (9.4%)	0.01
	CR	0	52 (13.3%)		0	7 (10.9%)	
	PR	39 (76.5%)	245 (62.8%)		39 (76.5%)	42 (65.6%)	
	SD	11 (21.6%)	40 (10.3%)		11 (21.6%)	9 (14.1%)	
	PD	1 (2.0%)	12 (3.1%)		1 (2.0%)	0	

Surgical approach	Minimally invasive surgery	12 (7.4%)	136 (8.1%)	0.46	12 (7.4%)	20 (12.3%)	0.14
	Laparotomy	150 (92.6%)	1538 (91.9%)		150 (92.6%)	142 (87.7%)	

PNI: perineural invasion; FIGO: International Federation of Gynecology and Obstetrics; NACT: neoadjuvant chemotherapy; pCR: pathological complete remission; CR: complete remission; PR: partial remission; SD: stable disease; PD: progress disease.

**Table 2:** Postoperative characteristics and adjuvant therapy.

Parameters		PNI (+), *n* = 162	PNI (-), *n* = 162	*P*
LVSI	Yes	54 (33.3%)	51 (31.5%)	0.72
	No	108 (66.7%)	111 (68.5%)	

Ovarian invasion	Yes	8 (5.0%)	3 (1.9%)	0.30
	No	137 (84.6%)	143 (88.3%)	
	Ovary Preserved	17 (10.4%)	16 (9.8%)	

The lower segment of uterine invasion	Yes	38 (23.5%)	26 (16.1%)	0.09
	No	124 (76.5%)	136 (83.9%)	

Deep stromal of cervical canal invasion	Yes	107 (66.1%)	64 (39.5%)	0.01
	No	55 (33.9%)	98 (60.5%)	

Deep stromal of outer cervix invasion	Yes	147 (90.7%)	99 (61.1%)	0.01
	No	15 (9.3%)	63 (38.9%)	

Tunica adventitia of cervix invasion	Yes	73 (45.1%)	17 (10.5%)	0.01
	No	89 (54.9%)	145 (89.5%)	

Lymph node invasion	Yes	57 (35.2%)	28 (17.3%)	0.01
	No	105 (64.8%)	134 (82.7%)	

Positive margin	Yes	60 (37.0%)	24 (14.8%)	0.01
	No	102 (63.0%)	138 (85.2%)	

Postoperative adjuvant therapy	Radiation with or without chemotherapy	130 (80.2%)	112 (69.1%)	0.03
	Chemotherapy Alone	17 (10.5%)	26 (16.1)	
	Observation	15 (9.3%)	24 (14.8%)	

LVSI: lymphovascular space invasion.

## Data Availability

All the original data of this study have been uploaded to the database specified by the Ethics Committee of Sun Yat-sen University Cancer Center, RDD Management Committee, http://www.researchdata.org.cn/. Data are available upon reasonable request.

## Additional Points

*Precis*. (i) PNI is likely to occur in cervical cancer patients with risk factors for recurrence. (ii) PNI is related to worse overall survival among cervical cancer patients without high-risk factors. (iii) We prove PNI plays the role as an intermediate-risk factor in cervical cancer patients.

## Supplementary Materials

Supplementary Materials Figure 1S: flow diagram of recruitment and propensity score matching
