# Supplementary figures and images for: Perineural Invasion Should Be Regarded as an Intermediate-Risk Factor for Recurrence in Surgically Treated Cervical Cancer: A Propensity Score Matching Study
Source: Dis Markers. 2021 Aug 3;2021:1375123. doi: 10.1155/2021/1375123 (PMC8357507; doi:10.1155/2021/1375123)

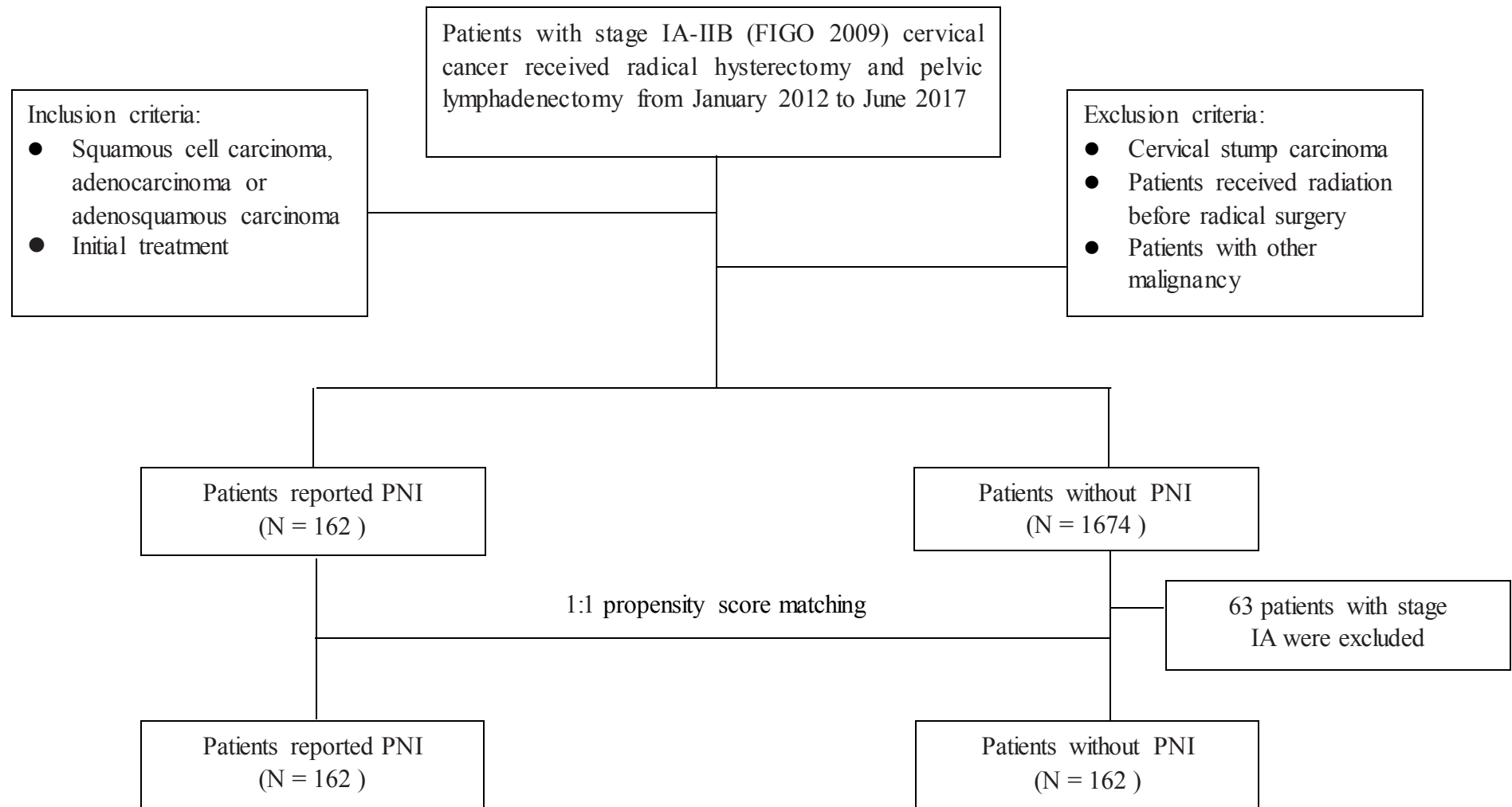

**Figure 1S.** Flow diagram of recruitment and propensity score matching

Supplement: Supplementary Materials — Figure 1S: flow diagram of recruitment and propensity score matching [file 1375123.f1.pdf]
